# Effect of the coronavirus pandemic on nutrition and health of adults in Calabar, Nigeria: A post-lockdown analysis

**DOI:** 10.4102/hsag.v27i0.1876

**Published:** 2022-08-17

**Authors:** Eridiong O. Onyenweaku, Hema Kesa, Alex K. Tchuenchieu, Anesu G. Kuhudzai

**Affiliations:** 1 Food Evolution and Research Laboratory, School of Tourism and Hospitality, College of Business and Economics, University of Johannesburg, Johannesburg, South Africa; 2 Centre for Food and Nutrition Research, Institute of Medical Research and Medicinal Plants Studies, Yaounde, Cameroon; 3 Statistical Consultation Services (STATKON), Post Graduate School, University of Johannesburg, Johannesburg, South Africa

**Keywords:** nutrition, health, food security, food frequency, NCDs, COVID-19, pandemic, Calabar

## Abstract

**Background:**

The food security and nutrition of millions of people around the world is currently being threatened by the coronavirus disease 2019 (COVID-19) pandemic, an evolving health crisis.

**Aim:**

To evaluate the effect of the COVID-19 pandemic on nutrition and health of adults in Calabar, especially after the hard lockdown.

**Setting:**

Online cross-sectional survey in Calabar, Nigeria.

**Method:**

After sample size determination, an online questionnaire was designed, content-validated by nutrition experts and piloted on 20 respondents. The questionnaire link was circulated for 6 weeks (April–May, 2021). The questionnaire was structured to gather socio-economic data, lifestyles of the participants (especially younger adults) and changes in dietary intake and health. Descriptive statistics and Pearson’s correlation were used to define the proportion of responses for each question and check for association.

**Results:**

No glaring nutrition or health problems was observed in the surveyed population (385 respondents), but many (50%) earned very low monthly income (< 50 000 naira). A drop in finances seemed to have indirectly caused a decrease in food consumption post-lockdown. A strong association between age and health risks was observed; similarly, alcohol intake was significantly affected by income and age.

**Conclusion:**

The pandemic caused many changes in people’s dietary habits and lifestyles, including financial setbacks. Apparently, education and proper enlightenment play a major role in food choices (despite limited resources), thus ensuring adequate nutrition and reducing health risks in the face of a pandemic.

**Contribution:**

This study has affirmed the efficacy of nutrition education and proper awareness in ensuring healthy dietary choices, optimal health and reduced risks of diseases.

## Introduction

Coronavirus disease 2019 (COVID-19), caused by the novel severe acute respiratory syndrome coronavirus 2 (SARS-CoV2) is a public health emergency that has significantly affected the world’s health and economy (Wiersinga et al. 2020). By 07 March 2021, the global death toll from COVID-19 had topped 2.5 million, with up to 116 million cases documented (World Health Organization [WHO] 2020). The COVID-19 emergency could have far-reaching implications for developing countries, where the combined effects of COVID-19 infection and mortality, unexpected consequences of corresponding mitigation measures and the looming global recession could affect nutrition and health (UN 2020). Coronavirus disease 2019 poses a health, social and economic threat to sub-Saharan Africa (SSA) (Ayanlade & Radeny 2020). This vulnerability is attributed to many factors, including poor health systems that hinder testing, timely detection and access to services for the treatment of COVID-19 (Gilbert et al. 2020; Madzorera et al. 2021).

The food security and nutrition of millions of people around the world are currently being threatened by the COVID-19 pandemic, which is an evolving health and human crisis (Onyenweaku et al. 2019). Hundreds of millions of people were already suffering from hunger and malnutrition before the COVID-19 pandemic, and unless immediate action is taken, there could be a global food emergency (FAO 2020). In the future, the combined effects of COVID-19 itself, as well as corresponding control measures and the emerging global recession, could disrupt the functioning of food systems if precautionary actions are not taken to forestall such an occurrence. Such disruption can result in serious health and nutrition consequences.

The COVID-19 pandemic control measures that have been put in place are already affecting global food supply chains. For instance, the travel bans and lockdowns are slowing down harvests in some parts of the world, leaving millions of seasonal workers without livelihoods, whilst also constraining the transport of food to markets (World Food Programme 2020). Farmers have been burying perishable produce as a result of supply chain disruption and falling consumer demand. Consequently, a lot of people in the urban areas now find it difficult to access fresh fruits and vegetables, dairy, meat and fish (UN 2020). It should be noted that the majority of Africa’s population takes its food from local markets (Bremner 2012).

The COVID-19 pandemic caused governments across the world to enforce movement restrictions or lockdowns and social distancing rules, in a bid to adhere to public health recommendations aimed at containing the spread of the disease (Hossain, Sultana & Purohit 2020). This has necessitated many changes in people’s lifestyles, ranging from reduced physical activities, more online activities, fluctuations in income and adjustments in eating habits, closure of offices and businesses, employees being asked to work from home except for essential services, whilst others have even lost their jobs (Bloch, Halle & Steinacker 2020). This has led to high levels of unemployment and loss of income, whilst on the other hand, the rising food costs are making access to food difficult for many. This can further worsen the existing problem of malnutrition.

In Nigeria, a country in SSA, the pandemic has caused many people (parents and sponsors inclusive) to lose their jobs and/or experience a drop in business revenue. This indirectly affects young dependents who get their daily subsistence and even school fees from their caretakers. Apart from affecting the quality of food available to such people, in the long run, this may pose some health risks to many. The incidence of malnutrition, especially undernutrition, may increase, and on the other hand, if the young people are kept at home from school for too long because of the pandemic, some may begin to experience conditions relating to overnutrition such as obesity or becoming overweight and even diabetes (Onyenweaku et al. 2019).

The lack of physical activity, smoking and alcohol consumption because of distress are often reported as population habits during this period, and these lifestyle habits are risk factors for noncommunicable diseases (NCDs) such as diabetes, hypertension and obesity (Cross, Babicz & Cushman 1994). Consequently, malnutrition, either in terms of quantity or quality cannot be ruled out. In fact, overeating and the consumption of unhealthy foods when staying at home for a long period are common, leading to people becoming overweight or obese (Tchuenchieu et al. 2021). On the other hand, many persons might change their eating habits, taking into consideration factors like food availability or prices (as a result of food insecurity).

This study therefore seeks to evaluate the effect of the COVID-19 lockdown on the dietary habits of young adults in Calabar, Nigeria. It also seeks to ascertain the health outcomes that changes in food consumption patterns of the study population may lead to, whether positive or negative.

## Research methods and design

### Study design

This was an online cross-sectional survey.

### Setting

The city of Calabar, which is the capital of Cross River state in southern Nigeria, was selected as the study area based on its unique population, dietary diversity and the availability of field workers for data collection. Calabar is an ancient city, consisting of a large and densely mixed population of about 600 000 people (Macrotrends 2021). The presence of higher educational institutions therein is responsible for a large number of young people who are following undergraduate and postgraduate programmes.

### Study population and sampling strategy

The population for the online cross-sectional study was adults residing in Calabar metropolis, whilst the sample population consisted of 385 respondents from the city of Calabar in Nigeria.

#### Sample size calculation

This study was carried out online for a period of 6 weeks, and a target sample size of 384 was calculated using the Leslie Kish formula to determine the sample size (Kish 1965):


n=Z×Z.p(1−p)/e×e [Eqn 1]

Where:

*n* = estimated sample size

*Z* = standard normal deviate usually set at 1.96 for 95% confidence

*P* = prevalence of disease under study put at 50% where prevalence is not ascertained

1 – *p* = 0.5

*e* = degree of accuracy desired, set at 0.05 substituting the above values, *n* = 384.

#### Sampling procedure

Random sampling was used to select the respondents alongside the snowball sampling method, bringing the total sample size to 385 participants. The questionnaire was circulated via an online survey link sent across to respondents electronically, as it was easier to gather data this way during this period because of the pandemic-related restrictions in movement and social distancing. The researchers and field workers forwarded the questionnaire link randomly to people on their e-mail and WhatsApp contact lists; those respondents in turn sent it out to their own networks (snowball sampling).

### Questionnaire design and data collection

A structured questionnaire was designed to gather information from respondents. The questionnaire was designed to collect information about the participants’ socio-economic status, health, dietary intake prior to and during the COVID-19 lockdown and lifestyle. The knowledge, attitude and practice (KAP) questionnaire was prepared with questions to test participants’ knowledge levels, attitudes and practices relating to nutrition and health, using a review of literature from research articles on similar studies. It was then converted into an online survey format using Google Forms, ensuring anonymity of the participants. The questionnaire was circulated electronically via the online survey link, which was sent across to participants via their e-mails and other social media platforms, such as WhatsApp. It was pretested on 20 respondents to ensure its reliability and validity before it was then circulated to others. The dietary choices and eating habits during the second (partial) lockdown were studied in order to bring out food security issues and ascertain food consumption patterns, particularly amongst young adults. The responses to the health-related questions were scored by allocating points to each health condition and lifestyle habit, such as smoking tobacco. A total score of 11 points was arrived at, with 11 signifying the highest health risk score.

#### Data analysis

The proportion of replies for each question and the total distribution in the total score of each questionnaire were defined using descriptive statistics such as frequencies, percentages and charts. The Statistical Package for Social Sciences (SPSS, version 20.0) and Microsoft Excel 2010 were used to conduct all statistical analyses. The mean and standard deviation (SD) of the values were calculated and published. The health risk scores of the respondents were calculated using a scoring system, and the scores were grouped into low (0–5), medium (6–8) and high (9–11). To assess for the association between health scores and sociodemographic variables, correlation analysis was done using the SPSS; this inversely determined the level of health risks of each group. Statistical significance was accepted as *p* < 0.05.

#### Informed consent and data privacy

Before moving on to the questionnaire, respondents were required to thoroughly read and comprehend the content summary. Participants in the survey were assured that the data would be used solely for research purposes during the informed consent process. According to Google’s privacy policy (https://policies.google.com/privacy?hl=en), participants’ responses were anonymous and confidential. It was not required for participants to reveal their identities or contact information. Participants could also cease participating in the study and leave the questionnaire page at any point before the submission process, and thus their responses would not be saved. Only the given ‘submit’ button was used to save responses. By completing the survey, participants acknowledged their voluntary consent to participate in this anonymous study. Participation was also voluntary.

### Ethical considerations

Ethical clearance to conduct this study was obtained from the College of Business & Economics Research Committee (CBERC) of the University of Johannesburg (reference number: 20STH04).

## Results

### Sociodemographic characteristics of the surveyed population

Findings will hereby be reported on the first 385 replies to the online survey on food security and dietary habits during the COVID-19 lockdown and lifestyle behaviours. Table 1 presents the sociodemographic characteristics of the surveyed population (385 people). The survey covered young adults residing in the Cross River state capital, Calabar, located in southern Nigeria. Women constituted 48% of this population, whilst men made up 42%; the remaining 10% preferred not to state their gender. The majority of the respondents were young people aged below 40 years (about 73%). They mainly had a tertiary education level (65%) and just a few had a secondary education level (31%) or less (3%). A higher percentage of them reported being single (62%), whilst the others were married (28%). Just 17% were living alone, the others in either 2–6 person households (68%) or above (15%). Only 6% earned an income above average (above 300 000 naira monthly), whilst over 50% earned a very low monthly income of less than 50 000 naira. Most of them either had an informal or private sector job (about 60%), and a significant number were unemployed (25%).

**TABLE 1:** Sociodemographic characteristics of the surveyed population (*N* = 385).

Variable	Subvariable	Frequency	Percentage
Sex	Female	185	48.05
	Male	163	42.34
	Prefer not to say	37	9.61
	Total	385	100.00
Age group	18–29 years	184	47.79
	30–39 years	112	29.09
	40–49 years	74	19.22
	50–59 years	9	2.34
	60 years and above	6	1.56
	Total	385	100.00
Marital status	Divorced	30	7.79
	Married	108	28.05
	Single	239	62.08
	Widowed	8	2.08
	Total	385	100.00
Education	No formal education	3	0.78
	Primary school	10	2.60
	Secondary (high) School	121	31.43
	Tertiary institution	251	65.19
	Total	385	100.00
Monthly income	0–50 000 naira	194	50.40
	51 000–150 000 naira	108	28.10
	151 000–300 000 naira	59	15.30
	Above 300 000 naira	24	6.20
	Total	385	100.00
Household size	Live alone	66	17.10
	2	98	25.50
	3–6	163	42.30
	More than 6	58	15.10
	Total	385	100.00
Employment sector	Public	61	15.80
	Private	107	27.80
	Informal	122	31.70
	Unemployed	95	24.70
	Total	385	100.00

### Participants’ nutrition ‘knowledge, attitude and practice’ in the coronavirus disease 2019 context

On a general note, the participants showed a good knowledge of COVID-19 and the role of nutrition in fighting infections (Table 2). A good number of the participants (75.1%) agreed that healthy diets and lifestyle play a role in the prevention of the COVID-19 infection; about 80% also knew more now about the COVID-19 infection than when the pandemic began in December 2019. Over 90% were able to correctly identify the major symptoms of COVID-19 to be fever, respiratory distress and dry cough; 80% included sneezing as a major symptom. In response to the role of diet in disease prevention, almost all the respondents admitted that healthy diets provide essential nutrients and strengthen the immune system (95%), and healthy diets fight disease-causing micro-organisms (80%). Looking at their dietary habits, 40% reported eating twice a day whilst about 60% ate three or more times a day. Of 80% of the participants who said they skip meals, 25% skipped breakfast, another 25% skipped any three meals, whilst 23% skipped lunch. With regard to their reasons for skipping meals, lack of time was a primary reason (71%), closely followed by other reasons such as fasting and no appetite (70%), as well as weight watching (65.5%).

**TABLE 2:** Participants’ nutrition knowledge, attitudes and practices in the coronavirus disease 2019 context.

Questions	Answers	Percentage responses (*N* = 385)
Do you know about the coronavirus pandemic (COVID-19)?	No	6.00
	Yes	80.20
	Not sure	13.80
What are the main clinical symptoms of COVID-19?	Fever	91.00
	Respiratory distress	92.00
	Dry cough	90.00
Does a healthy diet and lifestyle play a role in the prevention of COVID-19?	No	1.30
	Yes	75.10
	Not sure	23.60
The role of healthy diet in preventing infections and disease	Fights disease-causing organisms	88.00
	Provides essential nutrients and strengthens the immune system	95.00
	None of the above	36.00
How times do you have your meals per day?	Once	1.00
	Twice	40.00
	Thrice	44.00
	More than thrice	15.00
Do you skip meals?	No	20.00
	Yes	80.00
Which meals do you normally skip?	Breakfast	25.00
	Lunch	23.00
	Dinner	10.00
	All (anyone)	25.00
Most popular reason for skipping meals	Lack of time	71.20
	Others – fasting, no appetite	69.90
	Watching weight	65.50
	I prefer snacks to food*	58.20

COVID-19, coronavirus disease 2019.

### Changes in dietary pattern

**FIGURE 1:**
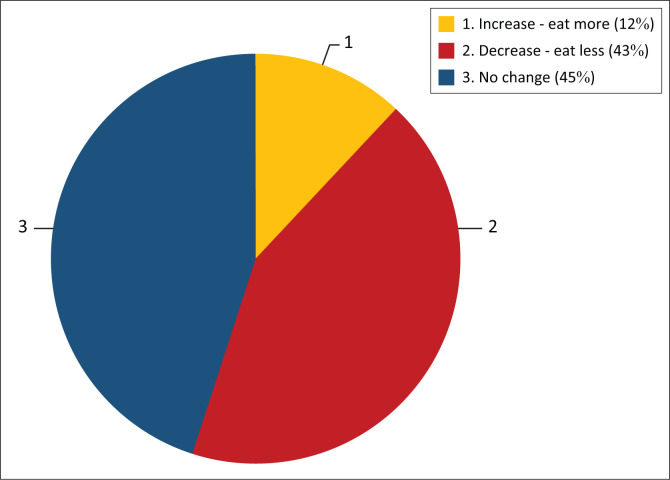
Change in quantity of food consumed.

**FIGURE 2:**
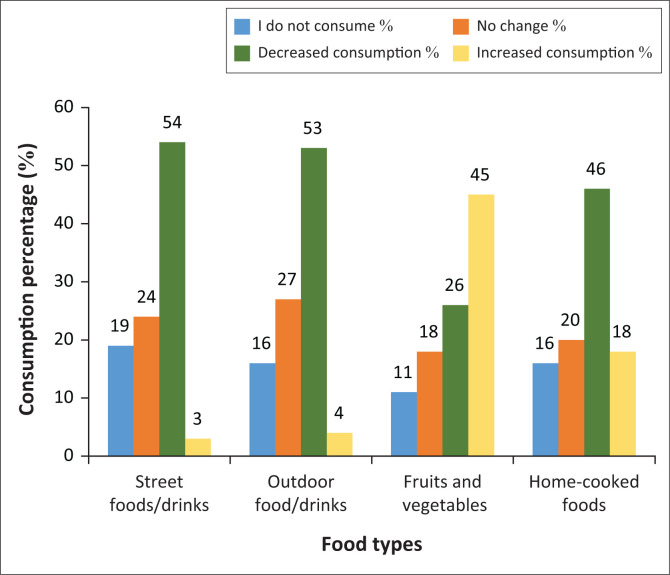
Changes in dietary consumption.

In comparing participants’ dietary patterns before the COVID-19 pandemic and presently (Figure 1), 43% reported a drop in their food intake, and 45% said there was no remarkable difference in their food consumption, whilst only 12% reported an increase. Figure 2 describes the changes in participants’ consumption of foods from various sources (such as homemade foods or street foods). It was also observed that 54% reported consuming less street food now than before; 24% reported that there was no change in their street food consumption. A similar trend was noticed in the consumption of ‘outdoor’ foods (i.e. foods not prepared at home, including fast food and restaurant meals). On the other hand, an opposite trend was observed with regard to the consumption of fresh foods (fruits and vegetables) – 45% consumed more of these now, and just 11% reported not consuming them at all. Similarly, 46% of the participants now had a higher preference for cooked foods, whilst 16% said there was no significant change. In response to whether government COVID-19 prevention measures affected their dietary choices, only 28% responded in the affirmative, and 38% said no whilst 34% were not sure.

### Participants’ perceptions of healthy eating or special diets consumed to prevent coronavirus disease 2019

In Table 3, a summary of participants’ perceptions of healthy eating is given. A qualitative evaluation of the responses given in relation to special diets consumed to prevent COVID-19 showed that vitamin C supplements were most consumed (over 50%), whilst fruits and vegetables such as oranges, ginger and watermelon followed closely (about 40%). Family and friends (80%), followed by social media (89%), were the popular means reported for getting information on these special COVID-19-preventive diets. Almost all (93%) who consumed these therapeutic substances agreed that the substances function by boosting the body’s immunity.

**TABLE 3:** Participants’ perceptions of a healthy diet.

Questions	Answers	Percentage responses (*N* = 385)
Has the knowledge you have gained during the pandemic positively affected your perception of certain foods?	No – My food choices have not changed	21.0
	Yes – I now make healthier food choices	68.3
	Indifferent	10.6
Consumption of green vegetables, onions, mushrooms, beans or peas and other micronutrient-rich foods can help boost the body’s immunity.	True	79.0
	False	2.6
	I do not know	18.4
On a general note, do you have access to the following:	Healthy balanced meals	88.3
	Enough fruits	79.5
	Enough vegetables	80.3
	Healthy snacks	70.4
Did the coronavirus pandemic affect your job or business adversely?	No	43.4
	Yes	25.5
	Not certain	31.1

### Change in health risk associated with coronavirus disease 2019 measures

The analysis of the surveyed population habits in relation to risk of noncommunicable diseases (Table 4a) showed that the majority of the people were not consuming alcohol (75.3%) or consumed alcohol less than 3 times a week (17.4%). A similar trend was observed regarding smoking (88.8% were nonsmokers and 51% of the smokers did so less than three times a week). Of the 385 surveyed young adults, only 3 were hypertensive, 5 were diabetic and 10 reported being diagnosed with a disease condition recently. Consequently, 68.8% of the respondents had a total health score of 11 (100%). The health scores were classified as follows: 0–5 low, 6–8 medium, 9–11 high; the higher the health score, the lower the health risk. The use of this scoring approach to assess the health risk level (Table 4b) identified age, marital status, education, number of people in the household and income as determining variables that affected the health risk factors significantly. Only gender and occupation did not significantly affect the health scores of the respondents. Most women (87%) and almost all who had a tertiary education (98.8%) had high health scores. In fact, the health risk tended to be higher (lower health score) amongst men, those living in a household of 3–6 persons and in those working in the informal sector. According to Table 4b, there was no significant difference based on gender (*p* = 0.120). It was also discovered using Pearson’s correlation that age and income significantly affected alcohol intake using the alcohol score values.

**TABLE 4a:** Lifestyle habits and health risks scores associated with noncommunicable diseases.

Questions	Answers	Frequency	Percentage
Do you consume alcohol?	No	290	75.3
	Yes	195	24.7
	Total	385	100.0
Weekly consumption of alcohol	Less than 3 times	97	49.7
	3–5 times	65	33.3
	More than 5 times	33	17.0
	Total	195	100.0
Do you smoke tobacco or cigarettes?	No	342	88.8
	Yes	43	11.2
	Total	385	100.0
Weekly smoking of tobacco or cigarettes	Less than 3 times	22	51.2
	3–5 times	17	39.5
	More than 5 times	4	9.3
	Total	43	100.0
Hypertensive?	No	382	99.2
	Yes	3	0.8
	Total	385	100.0
Diabetic?	No	380	98.7
	Yes	5	1.3
	Total	385	100.0
Any other recently diagnosed health condition?	No	375	97.4
	Yes	10	2.6
	Total	385	100.0

**TABLE 4b:** Change in lifestyle/associated health risk factors with sociodemographic variables.

Variables	Summary				Pearson chi-square		
	High	Low	Medium	Total	Value	*df*	Asymptotic Significance (two-sided)
**Gender**					7 324^a^	4	0.120
Female	161	4	20	185			
Male	127	9	27	163			
Prefer not to say	32	0	5	37			
**Age**					27 502^a^	8	0.001
18–29	166	0	18	184			
30–39	91	8	13	112			
40–49	51	5	18	74			
50–59	8	0	1	9			
60 and above	4	0	2	6			
**Marital status**					23 525^a^	6	0.001
Divorced	27	2	1	30			
Married	76	8	24	108			
Single	211	3	25	239			
Widowed	6	0	2	8			
**Education**					19 767^a^	6	0.003
No formal education	3	0	0	3			
Primary school	5	2	3	10			
Secondary (high) school	97	8	16	121			
Tertiary institution	215	3	33	251			
**Income**					28 818^a^	6	0.000
0–50 000 naira	178	0	16	194			
151 000–300 000 naira	41	6	12	59			
51 000–150 000 naira	83	5	20	108			
Above 300 000 naira	18	2	4	24			
**Household**					26 616^a^	6	0.000
2	93	0	5	98			
3–6	119	9	35	163			
Live alone	61	1	4	66			
More than 6	47	3	8	58			
**Occupation**					8 476^a^	6	0.205
Informal	102	7	13	122			
Private	87	2	18	107			
Public	47	3	11	61			
Unemployed	84	1	10	95			

*df*, degrees of freedom;

a , Pearson’s *P* value

## Discussion

The overall results of this study do not show any obvious nutrition or health problems in the surveyed population, which consisted of mainly young adults and few older adults. The results showed that participants were well aware of COVID-19 and dietary measures for the prevention of the disease. A glaring observation was the low income reported by many and reduction in food consumption, which is similar to the report of Matemilola and Elegbede (2017), which found about 33% of people in sub-Saharan Africa are suffering from undernutrition because of poor income levels. As this study population mainly comprised educated people (most with a tertiary education), they were still able to get a good food supply, possibly with support from their parents or sponsors as the case may be. This does not undermine the food insecurity challenge that exists in various parts of Africa, which has been worsened by the COVID-19 pandemic and its attendant control measures. Some of such persons may not have been covered by this online cross-sectional survey, which required Internet facilities and some level of literacy for participation (please note that many low-income earners are still able to own Android phones in Nigeria, especially amongst students, who constituted the main part of this study).

The surveyed population (being an enlightened one) showed very good knowledge of COVID-19 and its symptoms and preventive measures, correctly admitting the role of a healthy diet in boosting immunity and reducing the risk of infection. A similar case was reported for health workers in Nigeria by Okoro et al. (2021), where the surveyed population had a high KAP score of COVID-19 infection and prevention. This may be as a result of intensive and purposeful awareness campaigns successfully carried out in Nigeria to sensitise people on the pandemic (Akarika, Udo & Ikon 2020).

Most of the people reported generally eating twice or thrice a day, with lack of time being the major reason for skipping meals. With regard to changes in dietary pattern, the observed increase in consumption of fruits and vegetables alongside a reduction in the intake of street and outdoor foods and drinks is a commendable change, which could ultimately result in positive health outcomes. If sustained by this young population, this could reduce risks of NCDs in later life and increase life expectancy. Once again, this affirms the fact that knowledge can positively affect people’s practices. Muchiri, Gericke and Rheeder (2015) also confirmed that one way to promote healthy eating habits is via nutrition education. A decrease in the consumption of cooked meals by some respondents was observed, probably because of anxiety and lack of appetite during that difficult period and also people’s desire to consume more fruits and vegetables rather than their regular meals in order to strengthen their immune system. The WHO recommends that eating right and a healthy lifestyle with adequate physical activity are important for maintaining health and well-being (WHO 2020). This is important in the face of this pandemic, as reports show that mortality rates are higher amongst people living with comorbidities such as diabetes, hypertension and obesity (Narici et al. 2020).

Financial stability increases access to good nutrition. The surveyed population were mostly dependents who usually received financial support from parents, guardians or sponsors, hence their ability to eat well despite the low income levels reported by some of them. Most of them were dependents who were being sponsored by parents and guardians, and this also resulted in some of their negative responses to whether the pandemic affected their jobs. Globally, many people have lost jobs and businesses as a result of the challenges brought by the pandemic (Bloch et al. 2020).

Regarding lifestyle habits and health risks, the overall picture showed healthy habits, with most of the young people having low or no alcohol and tobacco intake. Only very few (fewer than 10 respondents) admitted to consuming alcohol or tobacco more than five times a week. In a recent opinion survey carried out in the United Kingdom, it was reported that about 33% of the respondents were taking steps to reduce or stop drinking, whilst 6% had stopped completely (Holmes & Angus 2021). During the peak of the pandemic, a slight contrast was reported by the Foundation for Alcohol Research and Education (FARE 2020), which found that 20% of respondents reported buying more alcohol than usual during the COVID-19 pandemic, with 70% of Australians drinking more than usual since the COVID-19 outbreak. Aside from depression, an assumption given for this increase in alcohol intake was the myth that alcohol kills COVID-19. This myth has now been debunked with the progress in understanding the COVID-19 infection. It was observed that age and income significantly affected alcohol intake using the alcohol score values. The female respondents had higher health scores and consequently lower health risks than the male respondents. This was because more men reported alcohol and tobacco intake and a few NCDs. It is important to educate people on the need to reduce alcohol and tobacco intake in order to improve health and prevent chronic diseases, especially as people age.

## Conclusion

The results of this study showed no significant nutrition and health issues amongst the assessed population, except for the very low income levels of most respondents, which may be as a result of the fact that most were young people who were still searching for proper jobs or still studying. This does not negate the fact that there are areas in Nigeria where people are experiencing food insecurity as a result of challenges posed by the COVID-19 pandemic and as such are in need of interventions. The dietary changes observed were good, with an increase in intake of fresh foods like fruits and vegetables. The effect of this was seen in the high health scores of most of the respondents. Consequently, there is a need for increased nutrition education to improve dietary choices and promote good health, especially amongst the less enlightened. This will go a long way at the national level to reduce the prevalence rate of disease and infection, increase life expectancy and productivity, especially in the face of this pandemic.

### Limitations of the study

This study was limited to an online survey, due to COVID-19 precautionary measures such as social distancing, which did not allow the researchers to move to certain semi-urban settings to include participants from those areas. Hence, this study only covers a particular socio-economic class of people who are educated and have access to Android phones and the Internet. The study was focused on the current nutrition and health situation of the young population during the second wave of the COVID-19 pandemic (in the first half of 2021). The validity of answers is also a general problem of online surveys that may be difficult to ascertain.
